# Relationship Between First 24-h Mean Body Temperature and Clinical Outcomes of Post-cardiac Surgery Patients

**DOI:** 10.3389/fcvm.2021.746228

**Published:** 2021-09-23

**Authors:** Fei Xu, Cheng Zhang, Chao Liu, Siwei Bi, Jun Gu

**Affiliations:** ^1^Department of Anesthesiology, The Affiliated Hospital, School of Medicine, Chengdu Women's & Children's Central Hospital, UESTC, Chengdu, China; ^2^West China of Medical School, Sichuan University, Chengdu, China; ^3^Department of Cardiovascular Surgery, West China Hospital, Sichuan University, Chengdu, China

**Keywords:** hypothermia, hyperthermia, clinical outcome, post-cardiac surgery, intensive care unit

## Abstract

**Background:** This study was aimed to investigate the relationship between first 24-h mean body temperature and clinical outcomes of post cardiac surgery patients admitted to intensive care unit (ICU) in a large public clinical database.

**Methods:** This is a retrospectively observational research of MIMIC III dataset, a total of 6,122 patients included. Patients were divided into 3 groups according to the distribution of body temperature. Multivariate cox analysis and logistic regression analysis were used to investigate the association between abnormal temperature, and clinical outcomes.

**Results:** Hypothermia (<36°C) significantly associated with increasing in-hospital mortality (HR 1.665, 95%CI 1.218–2.276; *p* = 0.001), 1-year mortality (HR 1.537, 95% CI 1.205–1.961; *p* = 0.001), 28-day mortality (HR 1.518, 95% CI 1.14–2.021; *p* = 0.004), and 90-day mortality (HR 1.491, 95% CI 1.144–1.943; *p* = 0.003). No statistical differences were observed between short-term or long-term mortality and hyperthermia (>38°C). Hyperthermia was related to the extended length of ICU stay (*p* < 0.001), and hospital stay (*p* < 0.001).

**Conclusion:** Hypothermia within 24h after ICU admission was associated with the increased mortality of post cardiac surgery patients. Enhanced monitoring of body temperature within 24h after cardiac surgery should be taken into account for improving clinical outcomes.

## Introduction

The management of body temperature (BT) was very important to the recovery of postoperative patients, especially for critically ill patients who have relatively severe physical conditions. Almost 57.1% patients were exposed to hypothermia (BT <36.0°C) among the patients admitted to intensive care unit (ICU) after operation (1) which claimed to be associated with mortality (2). Postoperative hyperthermia might be relevant to adverse clinical outcomes (3), and infection (4) in critically ill patients. Previous studies had indicated general anesthesia and surgical influence disturbed normal temperature regulation mechanism (5). Without preventative actions, influence of abnormal body temperature could be exaggerated with serious consequences.

As a special operation type, cardiac surgery had a great impact on circulation and physiology, and also brought enormous challenges to reduce mortality. Hypothermia and hyperthermia might occur after cardiac surgery attributed to long-term anesthesia and surgery exposure, the failure of the temperature maintenance, special hypothermia treatment and inflammatory response, and so on. Hyperthermia after cardiovascular surgery with cardiopulmonary bypass (CPB) could cause cognitive decline (3, 6). Grocott and colleagues revealed that postoperative high fever was associated with cognitive impairment at 6 weeks after coronary artery bypass grafting (6). Besides, there was study reporting that hypothermia effectively promoted endotoxin release through ischemic intestinal mucosa and had bad effects on brain tissue (7). Abnormal body temperature is disadvantageous to the prognosis of patients.

There were few studies explored the relationship between body temperature and adverse outcomes of patients after cardiac surgery, and no unified conclusion was obtained. Body temperature at single point in time raised bias risk inevitably (8), and could not fully reflect the overall effect of early postoperative body temperature. The object of this study was to investigate the association between first 24-h mean body temperature and clinical outcomes of post cardiac surgery patients admitted to ICU in a large public clinical database.

## Materials and Methods

### Data Source

The study is a retrospective study with data collected from Medical Information Mart for Intensive Care-III (MIMIC-III) database (9, 10). This is a large intensive care database open to the public including more than 40,000 patients admitted to ICU from Beth Israel Deaconess Medical Center (Boston, Massachusetts, USA) between 2001 and 2012. Beth Israel Deaconess Medical Center and the Beth Israel Deacon Medical Center's institutional review board approved the application of the database (approval code 40043439) and personal informed consent was abandoned.

### Study Population

Patients with cardiac surgery were identified using current procedural terminology numbered from 33,010 to 37,799. 18 to 89 years old patients were enrolled into the study. If patients were admitted to hospital or ICU multiple times, only the first record was analyzed. Patients stayed ICU <24h were excluded. Based on the attribution of temperature and existing research (11), Patients were divided into 3 groups: hypothermia group (BT <36.0°C), normal group (36.0°C ≤ BT ≤ 38.3°C), hyperthermia group (BT > 38.3°C).

### Data Collection and Definitions

The data was extracted from database by using structure query language (PostgreSQL, version 9.4.6, www.postgresql.org),and the codes in MIMIC Code Repository (https://github.com/MIT-LCP/mimic-website). Variables of this study included demographics, comorbidities, scoring systems, laboratory tests, and vital signs. Calculate the average of vital signs and laboratory tests with multiple results within 24h after ICU admission. Data analysis excluded variables with missing values exceeding 30% to avoid potential bias. Multiple imputation method was used to process variables with missing values <30%.

### Outcomes

The primary outcomes were in-hospital and 1-year mortality. The secondary outcomes included survival time, 28-day mortality, 90-day mortality, length of hospital stay and ICU stay, the intervention of continuous renal replacement therapy (CRRT) within 24h admitted to ICU, the incidence of acute kidney injury (AKI) within 7 days after ICU admission. The diagnosis of AKI was confirmed following the Kidney Disease: improving global outcomes (KDIGO) guideline (12).

### Statistical Analysis

Most continuous variables in this research were checked to be non-normally distributed and described as medians with interquartile ranges (IQRs), the rest were reported as mean ± standard deviation. Categorical variables were described as number and rate. Comparisons were performed with Kruskal-Wallis test or Mann-Whitney U or Welch's *t* test for continuous variables and chi-square or Fisher's exact tests for categorical variables. Propensity score matching (PSM) (13) was performed to adjust the imbalance of baseline between different groups. All selected variables were analyzed by univariate analysis firstly. Multivariate cox analysis or multivariate logistic regression analysis were performed to investigate relationships between abnormal temperature and clinical outcomes through adjusting confounding factors post-matched which had been analyzed in univariate analysis models with *P* < 0.05. Kaplan-Meier survival curves of 1 year mortality in different groups were constructed and compared by the log-rank test.

Data cleaning, statistical analyses and illustrations were conducted by using SPSS software version 24.0 (IBM Corporation, Armonk, NY, USA) and R software (version 4.0.3) consisted of “tableone” (14), “ggplot2” (15), “survival” (16), “survminer” (17), “lubridate” (18), “tidyverse” (19). *P* < 0.05 was considered statistically significant.

## Result

### Baseline Characteristics

**Figure 1:**
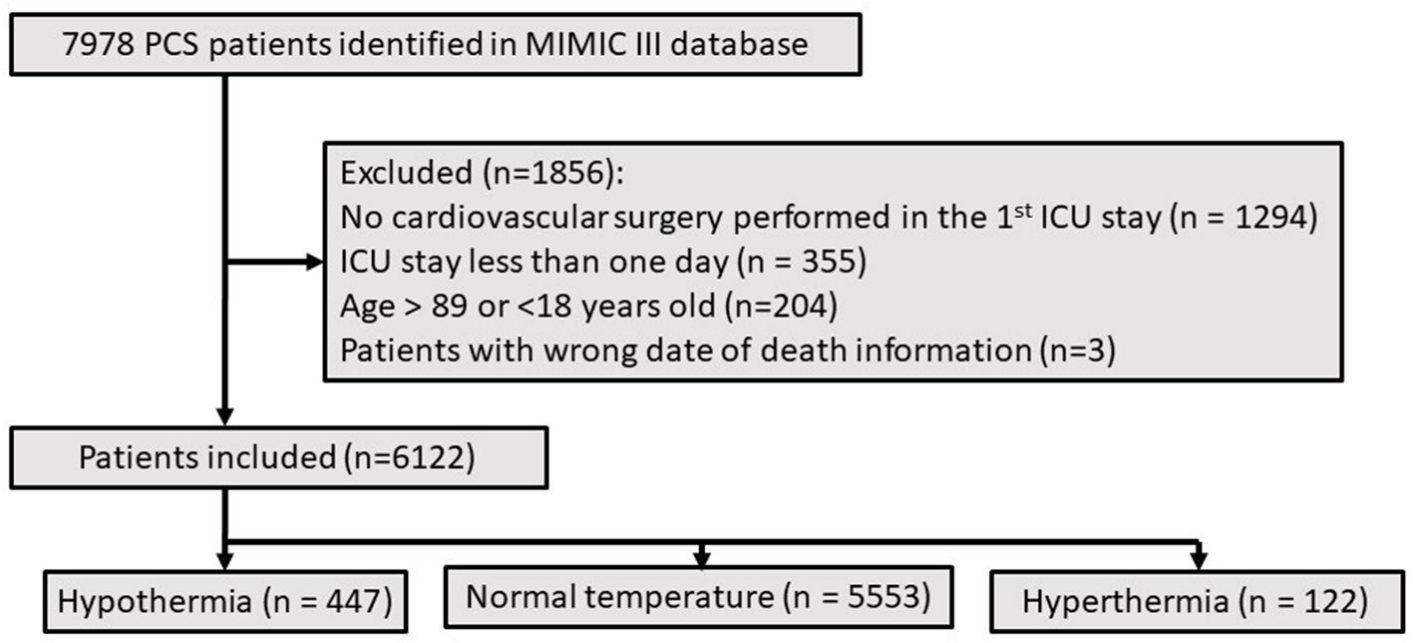
Flowchart of study cohort selection.

**Figure 2:**
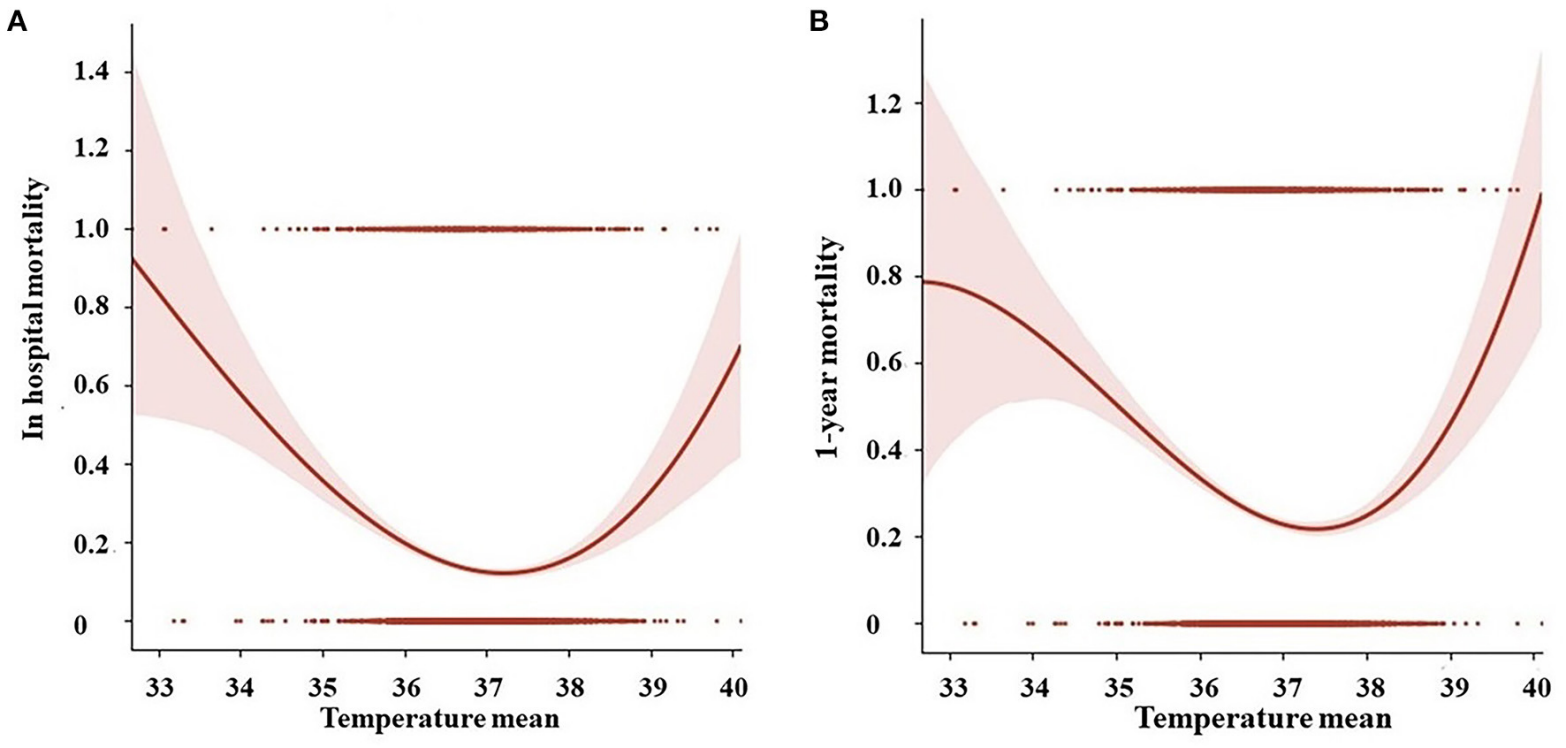
The relationship between **(A)** body temperature and in-hospital mortality; **(B)** body temperature and 1-year mortality by Polynomial regression.

**Figure 3:**
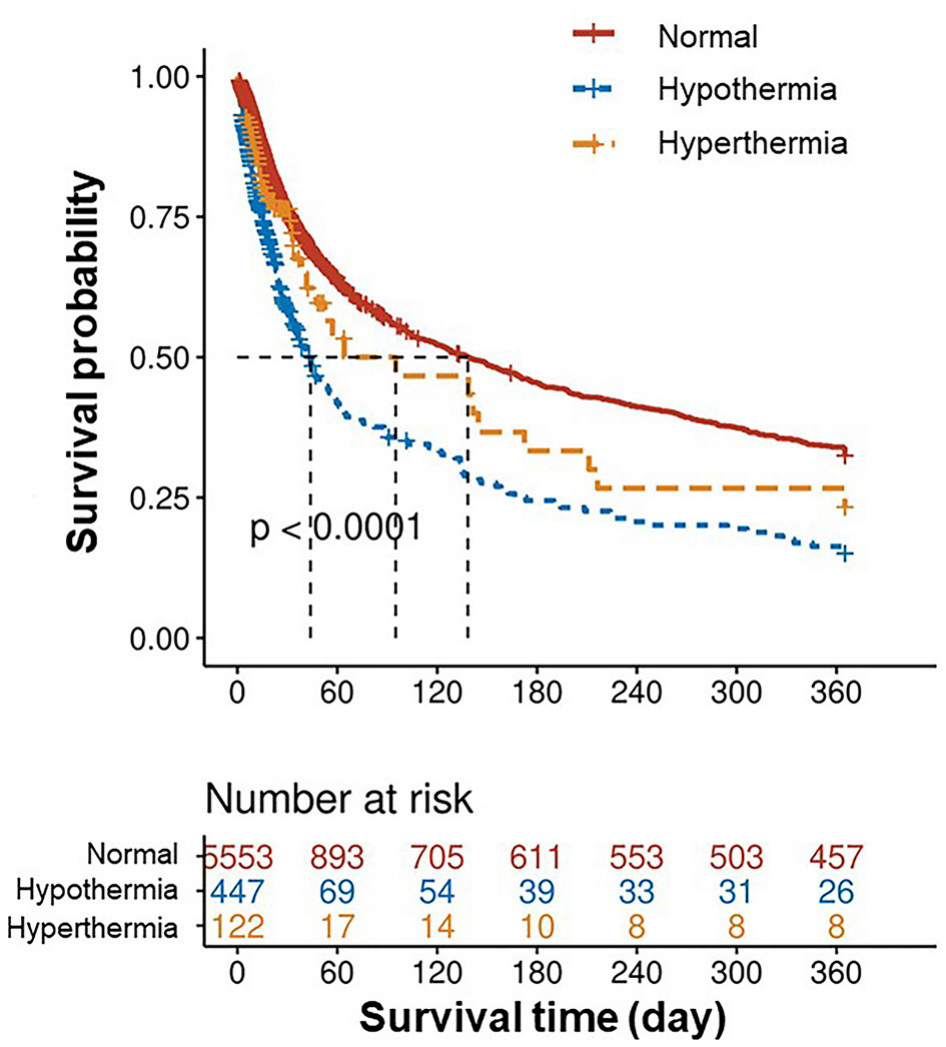
Kaplan-Meier curves of 1-year mortality by temperature category.

There were 6,122 patients concluded in our study (Figure 1), 447 patients (7.3%) belonged to hypothermia, 122 patients (2.0%) belonged to hyperthermia, 5,553 patients (90.7%) belonged to normal group. Clinical and laboratory test baseline characteristics of the populations were reported in Table 1. The association between body temperature and in-hospital mortality was described in Figure 2A (95% CI −0.043, −0.016, *p* < 0.001). The relationship between body temperature and 1-year mortality was shown in Figure 2B (95% CI −0.065, −0.032, *p* < 0.001). There were statistical differences between body temperature distribution of first 24- h after ICU admission and long-term mortality, that was described in Figure 3 (log-rank *p* < 0.001).

**Table 1:** Clinical and laboratory baseline characteristics of the overall cohort.

**Characteristics**	**Over all (*n* = 6,122)**	**Low temperature group (*n* = 447)**	**Normal temperature group (*n* = 5,553)**	**High temperature group (*n* = 122)**	***p-*value**
Temperature, median [IQR]	36.81 [36.43, 37.24]	35.8 [35.56, 35.9]	36.86 [36.52, 37.25]	38.58 [38.43, 38.71]	<0.001
Age, median [IQR]	66 [55, 76]	68.0 [59.0, 78.0]	66.0 [55.0, 76.0]	54.0 [40.0, 66.0]	<0.001
BMI, median [IQR]	27.84 [24.39, 31.84]	27.14 [24.21, 31.18]	27.83 [24.37, 31.79]	30.78 [26.7, 35.91]	<0.001
Male gender, n (%)	3,704 (60.503)	259 (57.942)	3,364 (60.580)	81 (66.393)	0.222
Admission type, *n* (%)					
Elective	1,535 (25.074)	100 (22.371)	1,429 (25.734)	6 (4.918)	<0.001
Emergency	4,487 (73.293)	342 (76.510)	4,031 (72.591)	114 (93.443)	
Urgent	100 (1.633)	5 (1.119)	93 (1.675)	2 (1.639)	
**Comorbidity**, ***n*** **(%)**					
Drug abuse, *n* (%)	208 (3.398)	14 (3.132)	186 (3.350)	8 (6.557)	0.146
Alcohol abuse, *n* (%)	474 (7.743)	44 (9.843)	414 (7.455)	16 (13.115)	0.016
Deficiency anemias, *n* (%)	1 340 (21.888)	94 (21.029)	1 209 (21.772)	37 (30.328)	0.070
Rheumatoid arthritis, *n* (%)	182 (2.973)	13 (2.908)	165 (2.971)	4 (3.279)	0.977
Metastatic cancer, *n* (%)	214 (3.496)	12 (2.685)	199 (3.584)	3 (2.459)	0.500
Liver disease, *n* (%)	463 (7.563)	52 (11.633)	408 (7.347)	3 (2.459)	<0.001
Renal failure, *n* (%)	869 (14.195)	81 (18.121)	769 (13.848)	19 (15.574)	0.041
Diabetes uncomplicated, *n* (%)	1,448 (23.652)	97 (21.700)	1,324 (23.843)	27 (22.131)	0.546
Hypertension, *n* (%)	758 (12.382)	66 (14.765)	680 (12.246)	12 (9.836)	0.206
Peripheral vascular, *n* (%)	794 (12.970)	73 (16.331)	709 (12.768)	12 (9.836)	0.057
Pulmonary circulation, *n* (%)	258 (4.214)	28 (6.264)	224 (4.034)	6 (4.918)	0.072
Valvular disease, *n* (%)	254 (4.149)	17 (3.803)	233 (4.196)	4 (3.279)	0.820
Cardiac arrhythmias, *n* (%)	911 (14.881)	87 (19.463)	805 (14.497)	19 (15.574)	0.017
**Laboratory tests within 24h after ICU**					
*F* calcium mean, median [IQR]	1.13 [1.08, 1.18]	1.11 [1.06, 1.16]	1.13 [1.08, 1.18]	1.1[1.02, 1.14]	<0.001
*T* calcium mean, median [IQR]	8.1 [7.65, 8.6]	8.15 [7.7, 8.7]	8.1 [7.65, 8.6]	7.8 [7.2, 8.4]	<0.001
WBC mean, median [IQR]	11.93 [8.9, 15.55]	11.4 [7.84, 14.47]	11.97 [9.0, 15.6]	13.2 [8.43, 17.1]	0.004
BUN mean, median [IQR]	18.33 [13.0, 29.4]	23.5 [15.5, 42.0]	18.0 [13.0, 28.0]	22.5 [14.5, 38.0]	<0.001
PT mean, median [IQR]	14.65 [13.6, 16.2]	15.2 [13.8, 18.05]	14.6 [13.55, 16.1]	14.83 [13.6, 16.6]	<0.001
INR mean, median [IQR]	1.3 [1.2, 1.5]	1.35 [1.2, 1.75]	1.3 [1.2, 1.5]	1.33 [1.2, 1.6]	<0.001
PTT mean, median [IQR]	33.3 [28.65, 41.6]	37.4 [30.7, 49.5]	33.0 [28.55, 41.1]	33.0 [28.05, 40.07]	<0.001
K^+^ mean, median [IQR]	4.19[3.87,4.5]	4.22[3.87,4.56]	4.19[3.88,4.5]	4.04[3.7,4.4]	0.004
Platelet mean, median [IQR]	175.33 [130.33, 233.33]	161.67 [111.33, 217.0]	176.0 [132.0, 234.0]	199.5 [134.0, 269.0]	<0.001
Lactate mean, median [IQR]	1.93 [1.4, 2.7]	2.3 [1.52, 3.4]	1.9 [1.4, 2.6]	2.0 [1.25, 3.07]	<0.001
Hemoglobin mean, median [IQR]	10.15 [9.15, 11.4]	10.09 [9.1, 11.2]	10.15 [9.15, 11.4]	10.35 [9.28, 12.3]	0.080
Hematocrit mean, median [IQR]	30.0 [27.3, 33.58]	29.88 [27.0, 33.45]	30.0 [27.3, 33.57]	30.8 [27.82, 36.0]	0.261
Glucose mean, median [IQR]	130.57 [116.0, 150.89]	133.0 [117.0, 159.25]	130.17 [116.0, 150.0]	139.25 [118.67, 165.33]	<0.001
Creatinine mean, median [IQR]	0.95 [0.72, 1.37]	1.05 [0.8, 2.0]	0.93 [0.7, 1.33]	1.08 [0.85, 2.13]	<0.001
**Vital sign**					
Spo_2_ mean, median [IQR]	97.77 [96.52, 98.81]	97.85 [96.39, 98.96]	97.77 [96.56, 98.81]	96.96 [95.33, 98.39]	<0.001
Respirate mean, median [IQR]	18.12 [16.08, 21.17]	17.78 [15.92, 20.85]	18.08 [16.07, 21.03]	23.03 [19.81, 26.62]	<0.001
Mean bp mean, median [IQR]	75.11 [70.17, 81.36]	74.61 [69.46, 80.39]	75.16 [70.24, 81.38]	74.96 [69.63, 83.87]	0.121
Dias bp mean, median [IQR]	58.39 [53.15, 64.3]	58.11 [52.44, 63.88]	58.41 [53.18, 64.29]	58.96 [54.1, 66.07]	0.276
Sys bp mean, median [IQR]	112.86 [105.52, 122.26]	111.13 [102.96, 118.56]	112.97 [105.71, 122.54]	113.15 [104.58, 125.96]	<0.001
Heart rate mean, median [IQR]	85.83 [77.53, 96.52]	80.97 [71.7, 90.64]	86.0 [77.9, 96.5]	105.52 [92.33, 117.19]	<0.001
**Score system**					
SPAS ii, median [IQR]	38 [30, 48]	44.0 [34.0, 56.0]	37.0 [30.0, 47.0]	43.0 [33.0, 55.0]	<0.001
SOFA, median [IQR]	5 [3, 8]	6.0 [4.0, 9.0]	5.0 [3.0, 7.0]	7.0 [5.0, 10.0]	<0.001
**Outcome**					
Hospital interval, median [IQR]	9.91 [6.07, 17.69]	10.0 [5.94, 19.09]	9.85 [6.06, 17.27]	17.91 [10.14, 30.06]	<0.001
ICU interval, median [IQR]	3.34 [1.86, 8.14]	3.86 [2.03, 9.15]	3.28 [1.83, 7.86]	9.99 [4.51, 17.22]	<0.001
Survival time, median [IQR]	11.15 [6.26, 27.17]	12.12 [6.03, 31.47]	11.01 [6.25, 26.75]	20.03 [10.34, 33.56]	<0.001
AKI 7-day, n (%)	4,563 (74.534)	360 (80.537)	4,094 (73.726)	109 (89.344)	<0.001
CRRT, n (%)	299 (4.884)	38 (8.501)	252 (4.538)	9 (7.377)	<0.001
Death in hospital, n (%)	925 (15.109)	133 (29.754)	763 (13.740)	29 (23.770)	<0.001
Death 28-day, n (%)	1,114 (18.197)	153 (34.228)	925 (16.658)	36 (29.508)	<0.001
Death 90-day, n (%)	1,301 (21.251)	174 (38.926)	1,089 (19.611)	38 (31.148)	<0.001
Death1-year, n (%)	1,606 (26.233)	201 (44.966)	1,362 (24.527)	43 (35.246)	<0.001

*BMI, body mass index; F calcium, free calcium; T calcium, total calcium; WBC, white blood cell count; BUN, Blood urea nitrogen; PT, prothrombin time; INR, international normalized ratio; PTT, partial thromboplastin time; SOFA, sequential organ failure assessment; SPAS, simplified acute physiology score; AKI, acute kidney injury; CRRT, continuous renal replacement therapy; ICU, the intensive care unit; SPO2, pulse oxygen saturation; BT, body temperature; Data are represented as median (interquartile range) or n (%), mean (SD, Standard Deviation)*.

### Hypothermia

The comparison between hypothermia and normal group was performed before (Supplemental Table 1) and after (Supplemental Table 2, Table 2) PSM. There were no significant differences in hospital interval, ICU interval and survival time (Table 2) across univariate analysis. After adjusting potential confounding factors, the results of multivariate cox analysis and multivariate logistic regression analysis were that in-hospital mortality (HR 1.665, 95% CI 1.218–2.276; *p* = 0.001) and 1-year mortality (HR 1.537, 95% CI 1.205–1.961; *p* = 0.001) significantly increased in hypothermia group. There were higher 28-day mortality (HR 1.518, 95% CI 1.14–2.021; *p* = 0.004) and 90-day mortality (HR 1.491, 95% CI 1.144–1.943; *p* = 0.003) compared to normal group. The incidence of AKI within 7-day and intervention of CRRT within the first day after ICU admission were both showed no significant differences between 2 groups (Table 3).

**Table 2:** Post-PSM matched between hypothermia and normal group.

	**Over all (*n* = 894)**	**Normal (*n* = 447)**	**Hypothermia (*n* = 447)**	***p*-value**
**Laboratory tests within 24h after ICU**				
*F* calcium mean, median [IQR]	1.12 [1.07, 1.18]	1.13 [1.09, 1.19]	1.11 [1.06, 1.16]	<0.001
*T* calcium mean, median [IQR]	8.1 [7.67, 8.7]	8.1 [7.65, 8.6]	8.15 [7.7, 8.7]	0.281
WBC mean, median [IQR]	11.47 [8.37, 15.05]	11.57 [8.8, 15.4]	11.4 [7.84, 14.47]	0.068
BUN mean, median [IQR]	20.0 [14.5, 35.25]	18.5 [14.0, 30.67]	23.5 [15.5, 42.0]	<0.001
PT mean, median [IQR]	14.97 [13.8, 17.1]	14.7 [13.8, 16.4]	15.2 [13.8, 18.05]	0.003
INR mean, median [IQR]	1.35 [1.2, 1.6]	1.3 [1.2, 1.5]	1.35 [1.2, 1.75]	0.006
PTT mean, median [IQR]	35.3 [29.75, 44.9]	33.8 [29.1, 41.9]	37.4 [30.7, 49.5]	<0.001
K^+^ mean, median [IQR]	4.2 [3.87, 4.53]	4.2 [3.89, 4.51]	4.22 [3.87, 4.56]	0.649
Platelet mean, median [IQR]	166.0 [121.0, 223.0]	169.0 [130.0, 226.33]	161.67 [111.33, 217.0]	0.018
Lactate mean, median [IQR]	2.1 [1.45, 3.09]	2.0 [1.4, 2.8]	2.3 [1.52, 3.4]	<0.001
Hemoglobin mean median [IQR]	10.04 [9.07, 11.28]	10.0 [9.07, 11.37]	10.09 [9.1, 11.2]	0.789
Hematocrit mean median [IQR]	29.8 [27.2, 33.46]	29.52 [27.32, 33.43]	29.88 [27.0, 33.45]	0.886
Glucose mean median [IQR]	132.6 [116.29, 153.0]	131.7 [115.62, 150.0]	133.0 [117.0, 159.25]	0.166
Creatinine mean median [IQR]	1.0 [0.75, 1.6]	0.95 [0.73, 1.4]	1.05 [0.8, 2.0]	<0.001
**Score system**				
SPAS ii, median [IQR]	41 [32, 53]	38.0 [31.0, 49.0]	44.0 [34.0, 56.0]	<0.001
SOFA, median [IQR]	6 [4, 8]	5.0 [3.0, 8.0]	6.0 [4.0, 9.0]	<0.001
**Vital sign**				
Spo_2_ mean, median [IQR]	97.79 [96.46, 98.95]	97.78 [96.56, 98.9]	97.85 [96.39, 98.96]	0.859
Temperature mean, median [IQR]	35.99 [35.8, 36.86]	36.86 [36.52, 37.2]	35.8 [35.56, 35.9]	<0.001
Respirate mean, median [IQR]	17.88 [15.97, 20.93]	18.05 [16.07, 20.95]	17.78 [15.92, 20.85]	0.359
Mean bp mean, median [IQR]	74.71 [69.71, 80.35]	74.84 [70.1, 80.23]	74.61 [69.46, 80.39]	0.592
Dias bp mean, median [IQR]	57.91 [52.33, 63.58]	57.55 [52.19, 63.19]	58.11 [52.44, 63.88]	0.530
Sys bp mean, median [IQR]	112.11 [104.14, 120.28]	113.08 [105.27, 122.84]	111.13 [102.96, 118.56]	<0.001
Heart rate mean, mean (SD)	83.53 [74.5, 93.04]	86.21 [78.3, 95.29]	80.97 [71.7, 90.64]	<0.001
**Outcomes**				
Hospital interval, median [IQR]	9.97 [6.03, 17.97]	9.9 [6.33, 17.04]	10.0 [5.94, 19.09]	0.873
ICU interval, median [IQR]	3.77 [2.0, 8.7]	3.47 [1.98, 8.11]	3.86 [2.03, 9.15]	0.621
Survival time, median [IQR]	11.8 [6.31, 30.0]	11.7 [6.97, 29.29]	12.12 [6.03, 31.47]	0.341
Death in hospital, *n* (%)	205 (22.931)	72 (16.107)	133 (29.754)	<0.001
CRRT, *n* (%)	59 (6.600)	21 (4.698)	38 (8.501)	0.022
AKI 7-day, *n* (%)	689 (77.069)	329 (73.602)	360 (80.537)	0.014
Death 28-day, *n* (%)	241 (26.957)	88 (19.687)	153 (34.228)	<0.001
Death 90-day, *n* (%)	280 (31.320)	106 (23.714)	174 (38.926)	<0.001
Death 1-year, *n* (%)	329 (36.801)	128 (28.635)	201 (44.966)	<0.001

*F calcium, free calcium; T calcium, total calcium; WBC, white blood cell count; BUN, Blood urea nitrogen; PT, prothrombin time; INR, international normalized ratio; PTT, partial thromboplastin time; SOFA, sequential organ failure assessment; SPAS, simplified acute physiology score; AKI, acute kidney injury; CRRT, continuous renal replacement therapy; ICU, the intensive care unit; SPO2, pulse oxygen saturation; BT, body temperature; Data are represented as median (interquartile range) or n (%), mean (SD, Standard Deviation)*.

**Table 3:** Multivariable cox regression and logistic regression analysis for hypothermia.

	**<36.0°C (Pre-matched)**			**<36.0°C (Post-matched)**		
	**HR/OR**	**95%Cl**	***P*-value**	**HR/OR**	**95%Cl**	***p*-value**
Death in-hospital	1.475	1.204–1.806	<001	1.665	1.218 – 2.276	0.001
Death 1-year	1.409	1.199–1.655	<001	1.537	1.205–1.961	0.001
Death 28-day	1.387	1.15–1.674	0.001	1.518	1.14–2.021	0.004
Death 90-day	1.389	1.166–1.656	<001	1.491	1.144–1.943	0.003
AKI 7-day	0.871	0.521–1.417	0.588	1.094	0.762–1.573	0.627
CRRT	1.004	0.764–1.329	0.976	1.021	0.457–2.31	0.959

*AKI, acute kidney injury; CRRT, continuous renal replacement therapy; HR, hazard ratio; OR, odds ratio*.

### Hyperthermia

The comparison between hyperthermia and normal group was conducted before (Supplemental Table 3) and after (Supplemental Table 4, Table 4) PSM. The length of hospital stay and ICU stay were longer in hyperthermia group than normal group, respectively (*p* < 0.001,Table 4) across univariate analysis. After adjustment for potential confounding factors, there were no statistical differences in in-hospital mortality, 28-day mortality, 90-day mortality, 1-year mortality, the incidence of AKI within 7-day or the intervention of CRRT within the first day after ICU admission between hyperthermia group and normal group across multivariate analysis (Table 5).

**Table 4:** Post-PSM matched between hyperthermia and normal group.

	**Over all (*n* = 244)**	**Normal (*n* = 122)**	**Hyperthermia(*n* = 122)**	***p*-value**
**Laboratory tests within 24h after ICU**				
*F* calcium mean, median [IQR]	1.11 [1.06, 1.15]	1.12[1.08, 1.16]	1.1 [1.02, 1.14]	0.002
*T* calcium mean, median [IQR]	8.03 [7.4, 8.5]	8.2 [7.7, 8.55]	7.8 [7.2, 8.4]	0.003
WBC mean, median [IQR]	12.43 [8.77, 16.73]	11.85 [8.8, 16.05]	13.2 [8.43, 17.1]	0.617
BUN mean, median [IQR]	18.67 [13.0, 33.0]	17.0 [12.0, 24.67]	22.5 [14.5, 38.0]	0.005
PT mean, median [IQR]	14.57 [13.6, 16.4]	14.3 [13.4, 15.9]	14.83 [13.6, 16.6]	0.122
INR mean, median [IQR]	1.3 [1.2, 1.55]	1.3 [1.2, 1.5]	1.33 [1.2, 1.6]	0.113
PTT mean, median [IQR]	32.1 [27.9, 38.36]	30.7 [27.7, 37.65]	33.0 [28.05, 40.07]	0.104
K^+^ mean, median [IQR]	4.11 [3.75, 4.4]	4.14 [3.81, 4.41]	4.04 [3.7, 4.4]	0.146
Platelet mean, median [IQR]	202.5 [140.5, 267.0]	206.8 [147.0, 263.0]	199.5 [134.0, 269.0]	0.565
Lactate mean, median [IQR]	1.9[1.37, 2.68]	1.85 [1.4, 2.4]	2.0 [1.25, 3.07]	0.626
Hemoglobin mean, median [IQR]	10.5 [9.37, 12.3]	10.6 [9.5, 12.5]	10.35 [9.28, 12.3]	0.544
Hematocrit mean, median [IQR]	31.0 [27.62, 35.3]	31.1 [27.53, 35.03]	30.8 [27.82, 36.0]	0.657
Glucose mean, median [IQR]	136.2 [117.88, 157.75]	131.0 [117.25, 149.2]	139.25 [118.67, 165.33]	0.044
Creatinine mean, median [IQR]	1.0 [0.8, 1.65]	0.95 [0.7, 1.2]	1.08 [0.85, 2.13]	0.005
**Score system**				
SPAS ii, median [IQR]	37 [29, 49]	34.0 [25.0, 42.0]	43.0 [33.0, 55.0]	<0.001
SOFA, median [IQR]	6 [4, 9]	5.0 [2.0, 7.0]	7.0 [5.0, 10.0]	<0.001
**Vital sign**				
SpO_2_ mean, median [IQR]	97.59 [96.04, 98.9]	97.92 [96.7, 99.18]	96.96 [95.33, 98.39]	<0.001
Temperature mean, median [IQR]	38.31 [37.0, 38.58]	36.99 [36.64, 37.44]	38.58 [38.43, 38.71]	<0.001
Resp rate mean, median [IQR]	20.42 [17.3, 24.38]	17.82 [15.97, 21.29]	23.03 [19.81, 26.62]	<0.001
Mean bp mean, median [IQR]	76.14 [70.0, 84.69]	77.1 [70.58, 87.36]	74.96 [69.63, 83.87]	0.092
Dias bp mean, median [IQR]	59.91 [54.68, 67.32]	61.45 [55.07, 68.72]	58.96 [54.1, 66.07]	0.155
Sys bp mean, median [IQR]	114.83 [105.94, 125.07]	114.94 [108.25, 125.07]	113.15 [104.58, 125.96]	0.279
Heart rate mean, mean (SD)	97.326 (17.954)	90.644 (15.142)	104.008 (18.059)	<0.001
**Outcomes**				
Hospital interval, median [IQR]	14.18 [7.76, 24.93]	10.84 [6.25, 20.44]	17.91 [10.14, 30.06]	<0.001
ICU interval, median [IQR]	6.29 [2.58, 13.2]	3.74 [2.04, 9.01]	9.99 [4.51, 17.22]	<0.001
Survival time, median [IQR]	15.7 [7.9, 30.67]	12.25 [6.31, 24.2]	20.03 [10.34, 33.56]	0.001
CRRT, *n* (%)	14 (5.738)	5 (4.098)	9 (7.377)	0.271
AKI 7-day, *n* (%)	193 (79.098)	84 (68.852)	109 (89.344)	<0.001
Death in hospital, *n* (%)	46 (18.852)	17 (13.934)	29 (23.770)	0.050
Death 28-day, *n* (%)	55 (22.541)	19 (15.574)	36 (29.508)	0.009
Death 90-day, *n* (%)	62 (25.410)	24 (19.672)	38 (31.148)	0.040
Death 1-year, *n* (%)	67 (27.459)	24 (19.672)	43 (35.246)	0.006

*F calcium,free calcium; T calcium, total calcium; WBC, white blood cell count; BUN, Blood urea nitrogen; PT, prothrombin time; INR, international normalized ratio; PTT, partial thromboplastin time; SOFA, sequential organ failure assessment; SPAS, simplified acute physiology score; AKI, acute kidney injury; CRRT, continuous renal replacement therapy; ICU, the intensive care unit; SPO2, pulse oxygen saturation; BT, body temperature; Data are represented as median (interquartile range) or n (%), mean (SD, Standard Deviation)*.

**Table 5:** Multivariable cox regression and logistic regression analysis for hyperthermia.

	**>38.3°C (Pre-matched)**			**>38.3°C (Post-matched)**		
	**HR/OR**	**95%Cl**	***P*-value**	**HR/OR**	**95%Cl**	***P*-value**
Death in-hospital	1.001	0.686–1.46	0.995	1.061	0.492–2.29	0.88
Death 1-year	1.069	0.785–1.456	0.672	1.049	0.567–1.94	0.878
Death 28-day	1.068	0.761–1.499	0.704	1.126	0.561–2.259	0.739
Death 90-day	1.033	0.743–1.436	0.846	0.917	0.484–1.74	0.791
AKI 7-day	1.573	0.869–3.061	0.155	2.329	0.938–6.058	0.073
CRRT	0.684	0.251–1.644	0.427	3.909	0.656–34.76	0.164

*AKI, acute kidney injury; CRRT, continuous renal replacement therapy; HR, hazard ratio; OR, odds ratio*.

## Discussion

This study demonstrated hypothermia within first 24-h after ICU admission was significantly associated with the increased short-term mortality and long-term mortality of post-cardiac surgery patients. Otherwise, no statistical associations were observed between hyperthermia and mortality. Hyperthermia was related to the prolonged length of hospital stay and ICU stay. Incidence of acute renal injury and intervention of continuous renal replacement therapy were not associated with hypothermia or hyperthermia.

Previous study considered body temperature could be used to assess prognostic risk independently. Hypothermia increased one-year mortality and seemed more harmful than hyperthermia (20). This is in line with our results. The reason may be relative to the great physiological changes produced by hypothermia (such as left shift of oxygenation curve, decline of coagulation function and arrhythmia and so on), which may lead to aggravate tissue hypoxia, aggravation of multiple organ functions (21), and failure of fluid resuscitation (22). For patients who were diagnosed sepsis within 24h, hypothermia (<36.0°C) could increase 28-day mortality and 1-year mortality (23). Besides, hypothermia was also associated with the mortality of none-elderly sepsis patients (11). Moreover, patients whose bladder core temperature were <36°C after ICU admission associated with worse outcomes after coronary artery bypass grafting (CABG) under CPB (5). The results of these researches are consistent with ours. Contrary to our study, Jiwook Kim's study suggested hypothermia or hyperthermia were not interrelated with long-term mortality of severe surgery patients except cardiovascular surgery patients (24). This can be explained by the inconsistence at the key time of temperature. Owing to enlarge sample size in our study, association between the distribution of body temperature and long-term mortality was significant. Relative research confirmed that changes in body temperature could easily induce acute and further cardiovascular outcomes (25). Thus, temperature monitoring of critically ill patients is extremely necessary. Emphasizing the importance of temperature monitoring within first 24-h after ICU admission is a new sight to improve the clinical outcomes of post-cardiac surgery patients.

The effects of hyperthermia and hypothermia on human body were complicated (26, 27). Hyperthermia was likely a powerful factor in strengthening immunity to remove subsequent pathogen by itself (28). Infection (29) and systemic inflammatory response syndrome (SIRS) caused by CPB (30) both lead to hyperthermia after cardiac surgery (31). Recent study claimed that hyperthermia in first 24h can be used to diagnose inflammatory response and possible outcomes after CPB, in this study in-hospital mortality was not significantly different comparing SIRS with no SIRS patients, while length of ICU stay was longer in SIRS patients (30). Besides, earlier studies suggested hyperthermia was beneficial for some outcomes (23, 32) and did not improve 1-year mortality of post-cardiac surgery patients (24). In line with our research, relationship between hyperthermia and mortality was not statistically significant, but hyperthermia was related to the prolonged length of ICU and hospital stay.

The strength of our study is focusing on the impact of overall body temperature changes within 24h after cardiac surgery on clinical outcomes rather than emphasizing the adverse results associated with a single time point. This study may be of beneficial in alleviating the adverse outcomes of hypothermia in post-cardiac surgery patients. Our study also has some limitations. Firstly, number of hyperthermia samples is relatively small, enlarging sample size and multicenter database are necessary for verification in future. Secondly, method and site of body temperature measurement were not clarified in MIMIC-III database which could cause bias to results, more researches deserve to carry out to clarify details. Thirdly, this is a retrospective single center study, results should be explained with caution in other populations and regions.

## Conclusion

This retrospective observational study confirmed hypothermia within 24-h after ICU admission was associated with the elevated mortality of post-cardiac surgery patients while hyperthermia was not. The importance of temperature monitoring is worthy of attention to improve clinical outcomes after cardiac surgery in clinical treatment.

## Data Availability Statement

The original contributions presented in the study are included in the article/Supplementary Material, further inquiries can be directed to the corresponding author/s.

## Ethics Statement

Ethical review and approval was not required for the study on human participants in accordance with the local legislation and institutional requirements. Written informed consent for participation was not required for this study in accordance with the national legislation and the institutional requirements.

## Author Contributions

FX, CL, and SB gathered and processed the data. CL and CZ prepared the results. FX and SB contributed in writing the manuscript. JG put forward the idea and revised the manuscript. All authors have read and approved the final manuscript.

## Funding

Current project is supported by the Sichuan Science and Technology Program, China (Grant no. 2019YFS0352), Post-Doctor Research Project, West China Hospital, Sichuan University (20HXBH171).

## Conflict of Interest

The authors declare that the research was conducted in the absence of any commercial or financial relationships that could be construed as a potential conflict of interest.

## Publisher's Note

All claims expressed in this article are solely those of the authors and do not necessarily represent those of their affiliated organizations, or those of the publisher, the editors and the reviewers. Any product that may be evaluated in this article, or claim that may be made by its manufacturer, is not guaranteed or endorsed by the publisher.
