# Supplementary material for: Relationship Between First 24-h Mean Body Temperature and Clinical Outcomes of Post-cardiac Surgery Patients
Source: Front Cardiovasc Med. 2021 Sep 23;8:746228. doi: 10.3389/fcvm.2021.746228 (PMC8494946; doi:10.3389/fcvm.2021.746228)
Supplement: Supplementary file 1 [file Table_1.docx]

| **Supplemental table 1,** Pre-PSM matched between hypothermia and normal group | | | | |
| --- | --- | --- | --- | --- |
| Characteristics | Over all (n=6000) | Normal (n=5553) | Hypothermia (n=447) | p |
| Age, median [IQR] | 66[56,76] | 66.0[55.0,76.0] | 68.0[59.0,78.0] | **<0.001** |
| BMI, median [IQR] | 27.81[24.36,31.74] | 27.83[24.37,31.79] | 27.14[24.21,31.18] | 0.116 |
| Male gender, n (%) | 3623(60.383) | 3364(60.580) | 259(57.942) | 0.273 |
| Admission type, n (%) | | | | |
| ELECTIVE | 1529(25.483) | 1429(25.734) | 100(22.371) | 0.175 |
| EMERGENCY | 4373(72.883) | 4031(72.591) | 342(76.510) |  |
| URGENT | 98(1.633) | 93(1.675) | 5(1.119) |  |
| Comorbidity, n (%) | | | | |
| Drug abuse, n (%) | 200(3.333) | 186(3.350) | 14(3.132) | 0.805 |
| Alcohol abuse, n (%) | 458(7.633) | 414(7.455) | 44(9.843) | 0.067 |
| Deficiency anemias, n (%) | 1303(21.717) | 1209(21.772) | 94(21.029) | 0.714 |
| Rheumatoid arthritis, n (%) | 178(2.967) | 165(2.971) | 13(2.908) | 0.940 |
| Metastatic cancer, n (%) | 211(3.517) | 199(3.584) | 12(2.685) | 0.321 |
| Liver disease, n (%) | 460(7.667) | 408(7.347) | 52(11.633) | **0.001** |
| Renal failure, n (%) | 850(14.167) | 769(13.848) | 81(18.121) | **0.013** |
| Diabetes uncomplicated, n (%) | 1421(23.683) | 1324(23.843) | 97(21.700) | 0.305 |
| hypertension, n (%) | 746(12.433) | 680(12.246) | 66(14.765) | 0.120 |
| Peripheral vascular, n (%) | 782(13.033) | 709(12.768) | 73(16.331) | **0.031** |
| Pulmonary circulation, n (%) | 252(4.200) | 224(4.034) | 28(6.264) | **0.024** |
| Valvular disease, n (%) | 250(4.167) | 233(4.196) | 17(3.803) | 0.689 |
| Cardiac arrhythmias, n (%) | 892(14.867) | 805(14.497) | 87(19.463) | **0.005** |
| vasopressor, n (%) | 3354(55.900) | 3086(55.574) | 268(59.955) | 0.073 |
| Laboratory tests within 24h after ICU | | | | |
| Hematocrit mean, median [IQR] | 30.0[27.3,33.55] | 30.0[27.3,33.57] | 29.88[27.0,33.45] | 0.538 |
| Glucose mean, median [IQR] | 130.5[116.0,150.5] | 130.17[116.0,150.0] | 133.0[117.0,159.25] | **0.019** |
| Creatinine mean, median [IQR] | 0.95[0.7,1.35] | 0.93[0.7,1.33] | 1.05[0.8,2.0] | **<0.001** |
| F calcium mean, median [IQR] | 1.13[1.08,1.18] | 1.13[1.08,1.18] | 1.11[1.06,1.16] | **<0.001** |
| T calcium mean, median [IQR] | 8.1[7.67,8.6] | 8.1[7.65,8.6] | 8.15[7.7,8.7] | 0.085 |
| WBC mean, median [IQR] | 11.9[8.93,15.53] | 11.97[9.0,15.6] | 11.4[7.84,14.47] | **0.001** |
| BUN mean, median [IQR] | 18.0[13.0,29.0] | 18.0[13.0,28.0] | 23.5[15.5,42.0] | **<0.001** |
| PT mean, median [IQR] | 14.65[13.6,16.2] | 14.6[13.55,16.1] | 15.2[13.8,18.05] | **<0.001** |
| INR mean, median [IQR] | 1.3[1.2,1.5] | 1.3[1.2,1.5] | 1.35[1.2,1.75] | **<0.001** |
| PTT mean, median [IQR] | 33.3[28.7,41.6] | 33.0[28.55,41.1] | 37.4[30.7,49.5] | **<0.001** |
| K^+^ mean, median [IQR] | 4.2[3.88,4.5] | 4.19[3.88,4.5] | 4.22[3.87,4.56] | 0.199 |
| Platelet mean, median [IQR] | 175.0[130.33,233.0] | 176.0[132.0,234.0] | 161.67[111.33,217.0] | **<0.001** |
| Lactate mean, median [IQR] | 1.93[1.4,2.68] | 1.9[1.4,2.6] | 2.3[1.52,3.4] | **<0.001** |
| Hemoglobin mean, median [IQR] | 10.15[9.15,11.38] | 10.15[9.15,11.4] | 10.09[9.1,11.2] | 0.191 |
| Vent num, median [IQR] | 1[1,1] | 1.0[1.0,1.0] | 1.0[1.0,1.0] | 0.131 |
| SpO_2_ mean, median [IQR] | 97.77[96.56,98.82] | 97.77[96.56,98.81] | 97.85[96.39,98.96] | 1.000 |
| Vital sign | | | | |
| Temperature mean, median [IQR] | 36.8[36.42,37.21] | 36.86[36.52,37.25] | 35.8[35.56,35.9] | **<0.001** |
| Resp rate mean, median [IQR] | 18.07[16.06,21.02] | 18.08[16.07,21.03] | 17.78[15.92,20.85] | 0.095 |
| Mean bp mean, median [IQR] | 75.11[70.18,81.3] | 75.16[70.24,81.38] | 74.61[69.46,80.39] | 0.041 |
| Dias bp mean, median [IQR] | 58.38[53.13,64.25] | 58.41[53.18,64.29] | 58.11[52.44,63.88] | 0.307 |
| Sys bp mean, median [IQR] | 112.86[105.55,122.24] | 112.97[105.71,122.54] | 111.13[102.96,118.56] | **<0.001** |
| Heart rate mean, median [IQR] | 85.59[77.43,96.03] | 86.0[77.9,96.5] | 80.97[71.7,90.64] | **<0.001** |
| Score system | | | | |
| SPAS ii, median [IQR] | 38[30,48] | 37.0[30.0,47.0] | 44.0[34.0,56.0] | **<0.001** |
| SOFA, median [IQR] | 5[3,8] | 5.0[3.0,7.0] | 6.0[4.0,9.0] | **<0.001** |
| Outcome | | | | |
| Hospital interval, median [IQR] | 9.85[6.04,17.4] | 9.85[6.06,17.27] | 10.0[5.94,19.09] | 0.987 |
| Survival time, median [IQR] | 11.05[6.23,26.98] | 11.01[6.25,26.75] | 12.12[6.03,31.47] | 0.968 |
| ICU interval, median [IQR] | 3.3[1.84,7.9] | 3.28[1.83,7.86] | 3.86[2.03,9.15] | 0.061 |
| CRRT, n (%) | 290(4.833) | 252(4.538) | 38(8.501) | **<0.001** |
| AKI 7-day, n (%) | 4454(74.233) | 4094(73.726) | 360(80.537) | **0.002** |
| Death in hospital, n (%) | 896(14.933) | 763(13.740) | 133(29.754) | **<0.001** |
| Death 28-day, n (%) | 1078(17.967) | 925(16.658) | 153(34.228) | **<0.001** |
| Death 90-day, n (%) | 1263(21.050) | 1089(19.611) | 174(38.926) | **<0.001** |
| Death 1-year, n (%) | 1563(26.050) | 1362(24.527) | 201(44.966) | **<0.001** |
| BMI, body mass index; F calcium，free calcium; T calcium, total calcium; WBC, white blood cell count; BUN, Blood urea nitrogen; PT, prothrombin time; INR, international normalized ratio; PTT, partial thromboplastin time; SOFA sequential organ failure assessment; SPAS: simplified acute physiology score; AKI: acute kidney injury; CRRT: continuous renal replacement therapy; ICU: the intensive care unit; SPO2, pulse oxygen saturation; Data are represented as median (interquartile range) or n (%), mean (SD, Standard Deviation). | | | | |

| **Supplemental table 2**. Baseline of Post-PSM matched patients between hypothermia and normal group | | | | |
| --- | --- | --- | --- | --- |
| Characteristics | Over all (n=894) | Normal (n=447) | Hypothermia (n=447) | *P* |
| Age, median [IQR] | 68[59,79] | 68.0[59.0,78.0] | 68.0[59.0,78.0] | 0.719 |
| BMI, median [IQR] | 27.25[24.07,31.25] | 27.42[23.95,31.35] | 27.14[24.21,31.18] | 0.960 |
| Male gender, n (%) | 508(56.823) | 249(55.705) | 259(57.942) | 0.500 |
| Admission type, n (%) | | | | |
| ELECTIVE | 215(24.049) | 115(25.727) | 100(22.371) | 0.501 |
| EMERGENCY | 669(74.832) | 327(73.154) | 342(76.510) |  |
| URGENT | 10(1.119) | 5(1.119) | 5(1.119) |  |
| **Comorbidity, n (%)** | | | | |
| Drug abuse, n (%) | 27(3.020) | 13(2.908) | 14(3.132) | 0.845 |
| Alcohol abuse, n (%) | 76(8.501) | 32(7.159) | 44(9.843) | 0.150 |
| Rheumatoid arthritis, n (%) | 28(3.132) | 15(3.356) | 13(2.908) | 0.701 |
| Deficiency anemias, n (%) | 203(22.707) | 109(24.385) | 94(21.029) | 0.231 |
| Metastatic cancer, n (%) | 27(3.020) | 15(3.356) | 12(2.685) | 0.558 |
| Liver disease, n (%) | 111(12.416) | 59(13.199) | 52(11.633) | 0.478 |
| Renal failure, n (%) | 157(17.562) | 76(17.002) | 81(18.121) | 0.660 |
| Diabetes, n (%) | 196(21.924) | 99(22.148) | 97(21.700) | 0.872 |
| Hypertension, n (%) | 132(14.765) | 66(14.765) | 66(14.765) | 1.000 |
| Peripheral vascular, n (%) | 142(15.884) | 69(15.436) | 73(16.331) | 0.714 |
| Pulmonary circulation, n (%) | 52(5.817) | 24(5.369) | 28(6.264) | 0.568 |
| Valvular disease, n (%) | 39(4.362) | 22(4.922) | 17(3.803) | 0.413 |
| Cardiac arrhythmias, n (%) | 167(18.680) | 80(17.897) | 87(19.463) | 0.548 |
| BMI, body mass index | | | | |

| **Supplemental table 3**. Pre-PSM matched between hyperthermia and normal group | | | | |
| --- | --- | --- | --- | --- |
| **Characteristics** | Over all (n=5675) | Normal (n=5553) | Hyperthermia (n=122) | *p* |
| Age, median [IQR] | 66[55,76] | 66.0[55.0,76.0] | 54.0[40.0,66.0] | <0.001 |
| BMI, median [IQR] | 27.89[24.4,31.87] | 27.83[24.37,31.79] | 30.78[26.7,35.91] | <0.001 |
| Male gender, n (%) | 3445(60.705) | 3364(60.580) | 81(66.393) | 0.193 |
| ADMISSIONTYPE, n (%) | | | | |
| ELECTIVE | 1435(25.286) | 1429(25.734) | 6(4.918) | <0.001 |
| EMERGENCY | 4145(73.040) | 4031(72.591) | 114(93.443) |  |
| URGENT | 95(1.674) | 93(1.675) | 2(1.639) |  |
| **Comorbidity**, n (%) | | | | |
| Drug abuse, n (%) | 194(3.419) | 186(3.350) | 8(6.557) | 0.054 |
| Alcohol abuse, n (%) | 430(7.577) | 414(7.455) | 16(13.115) | 0.019 |
| Deficiency anemias, n (%) | 1246(21.956) | 1209(21.772) | 37(30.328) | 0.024 |
| Rheumatoid arthritis, n (%) | 169(2.978) | 165(2.971) | 4(3.279) | 0.843 |
| Metastatic cancer, n (%) | 202(3.559) | 199(3.584) | 3(2.459) | 0.507 |
| Liver disease, n (%) | 411(7.242) | 408(7.347) | 3(2.459) | 0.039 |
| Renal failure, n (%) | 788(13.885) | 769(13.848) | 19(15.574) | 0.586 |
| Diabetes uncomplicated, n (%) | 1351(23.806) | 1324(23.843) | 27(22.131) | 0.661 |
| hypertension, n (%) | 692(12.194) | 680(12.246) | 12(9.836) | 0.421 |
| Peripheral vascular, n (%) | 721(12.705) | 709(12.768) | 12(9.836) | 0.336 |
| Pulmonary circulation, n (%) | 230(4.053) | 224(4.034) | 6(4.918) | 0.624 |
| Valvular disease, n (%) | 237(4.176) | 233(4.196) | 4(3.279) | 0.616 |
| Cardiac arrhythmias, n (%) | 824(14.520) | 805(14.497) | 19(15.574) | 0.738 |
| **Laboratory tests within 24h after ICU** | | | | |
| Creatinine mean, median [IQR] | 0.95[0.7,1.35] | 0.93[0.7,1.33] | 1.08[0.85,2.13] | <0.001 |
| WBC mean, median [IQR] | 12.0[9.0,15.6] | 11.97[9.0,15.6] | 13.2[8.43,17.1] | 0.337 |
| BUN mean, median [IQR] | 18.0[13.0,28.5] | 18.0[13.0,28.0] | 22.5[14.5,38.0] | 0.005 |
| PT mean, median [IQR] | 14.6[13.57,16.1] | 14.6[13.55,16.1] | 14.83[13.6,16.6] | 0.174 |
| INR mean, median [IQR] | 1.3[1.2,1.5] | 1.3[1.2,1.5] | 1.33[1.2,1.6] | 0.076 |
| PTT mean, median [IQR] | 33.0[28.53,41.05] | 33.0[28.55,41.1] | 33.0[28.05,40.07] | 0.801 |
| K^+^ mean, median [IQR] | 4.19[3.87,4.5] | 4.19[3.88,4.5] | 4.04[3.7,4.4] | 0.002 |
| Platelet mean, median [IQR] | 176.5[132.0,235.0] | 176.0[132.0,234.0] | 199.5[134.0,269.0] | 0.072 |
| Lactate mean, median [IQR] | 1.9[1.4,2.6] | 1.9[1.4,2.6] | 2.0[1.25,3.07] | 0.753 |
| Hemoglobin mean, median [IQR] | 10.15[9.15,11.4] | 10.15[9.15,11.4] | 10.35[9.28,12.3] | 0.073 |
| Hematocrit mean, median [IQR] | 30.02[27.3,33.6] | 30.0[27.3,33.57] | 30.8[27.82,36.0] | 0.133 |
| Glucose mean, median [IQR] | 130.38[116.0,150.33] | 130.17[116.0,150.0] | 139.25[118.67,165.33] | 0.001 |
| F calcium mean, median [IQR] | 1.13[1.08,1.18] | 1.13[1.08,1.18] | 1.1[1.02,1.14] | <0.001 |
| T calcium mean, median [IQR] | 8.1[7.65,8.6] | 8.1[7.65,8.6] | 7.8[7.2,8.4] | <0.001 |
| **Vital sign** | | | | |
| Spo_2_ mean, median [IQR] | 97.76[96.53,98.81] | 97.77[96.56,98.81] | 96.96[95.33,98.39] | <0.001 |
| Temperature mean, median [IQR] | 36.87[36.53,37.29] | 36.86[36.52,37.25] | 38.58[38.43,38.71] | <0.001 |
| Respirate mean, median [IQR] | 18.14[16.09,21.21] | 18.08[16.07,21.03] | 23.03[19.81,26.62] | <0.001 |
| Mean bp mean, median [IQR] | 75.15[70.23,81.42] | 75.16[70.24,81.38] | 74.96[69.63,83.87] | 0.882 |
| Dias bp mean, median [IQR] | 58.42[53.21,64.34] | 58.41[53.18,64.29] | 58.96[54.1,66.07] | 0.228 |
| Sys bp mean, median [IQR] | 112.98[105.68,122.61] | 112.97[105.71,122.54] | 113.15[104.58,125.96] | 0.846 |
| Heartrate mean, median [IQR] | 86.2[78.05,97.05] | 86.0[77.9,96.5] | 105.52[92.33,117.19] | <0.001 |
| **Score system** | | | | |
| SPAS ii, median [IQR] | 37[30,48] | 37.0[30.0,47.0] | 43.0[33.0,55.0] | <0.001 |
| SOFA, median [IQR] | 5[3,7] | 5.0[3.0,7.0] | 7.0[5.0,10.0] | <0.001 |
| **Outcomes** | | | | |
| Hospital interval, median [IQR] | 9.9[6.09,17.61] | 9.85[6.06,17.27] | 17.91[10.14,30.06] | <0.001 |
| ICU interval, median [IQR] | 3.32[1.85,8.1] | 3.28[1.83,7.86] | 9.99[4.51,17.22] | <0.001 |
| Survival time, median [IQR] | 11.11[6.27,27.02] | 11.01[6.25,26.75] | 20.03[10.34,33.56] | <0.001 |
| CRRT, n (%) | 261(4.599) | 252(4.538) | 9(7.377) | 0.139 |
| AKI 7-day, n (%) | 4203(74.062) | 4094(73.726) | 109(89.344) | <0.001 |
| Death in hospital, n (%) | 792(13.956) | 763(13.740) | 29(23.770) | 0.002 |
| Death 28-day, n (%) | 961(16.934) | 925(16.658) | 36(29.508) | <0.001 |
| Death 90-day, n (%) | 1127(19.859) | 1089(19.611) | 38(31.148) | 0.002 |
| Death 1-year, n (%) | 1405(24.758) | 1362(24.527) | 43(35.246) | 0.007 |
| BMI, body mass index；F calcium，free calcium; T calcium, total calcium; WBC, white blood cell count; BUN, Blood urea nitrogen; PT, prothrombin time; INR, international normalized ratio; PTT, partial thromboplastin time; SOFA sequential organ failure assessment; SPAS: simplified acute physiology score; AKI: acute kidney injury; CRRT: continuous renal replacement therapy; ICU: the intensive care unit; SPO2, pulse oxygen saturation; Data are represented as median (interquartile range) or n (%), mean (SD, Standard Deviation). | | | | |

| **Supplemental table 4.** Baseline of Post-PSM matched patients between hyperthermia and normal group | | | | |
| --- | --- | --- | --- | --- |
| Characteristics | Over all (n=244) | Normal (n=122) | Hyperthermia (n=122) | P |
| Age, median [IQR] | 53[41,64] | 51.0[41.0,64.0] | 54.0[40.0,66.0] | 0.387 |
| BMI, median [IQR] | 30.75[26.04,35.81] | 30.23[25.16,35.6] | 30.78[26.7,35.91] | 0.581 |
| Male gender, n (%) | 164(67.213) | 83(68.033) | 81(66.393) | 0.785 |
| Admission type, n (%) | | | | |
| ELECTIVE | 14(5.738) | 8(6.557) | 6(4.918) | 0.859 |
| EMERGENCY | 226(92.623) | 112(91.803) | 114(93.443) |  |
| URGENT | 4(1.639) | 2(1.639) | 2(1.639) |  |
| **Comorbidity**, n (%) | | | | |
| Drug abuse, n (%) | 15(6.148) | 7(5.738) | 8(6.557) | 0.790 |
| Alcohol abuse, n (%) | 30(12.295) | 14(11.475) | 16(13.115) | 0.697 |
| Deficiency anemias, n (%) | 66(27.049) | 29(23.770) | 37(30.328) | 0.249 |
| Rheumatoid arthritis, n (%) | 5(2.049) | 1(0.820) | 4(3.279) | 0.175 |
| Metastatic cancer, n (%) | 5(2.049) | 2(1.639) | 3(2.459) | 0.651 |
| Liver disease, n (%) | 6(2.459) | 3(2.459) | 3(2.459) | 1.000 |
| Renal failure, n (%) | 31(12.705) | 12(9.836) | 19(15.574) | 0.178 |
| Diabetes, n (%) | 54(22.131) | 27(22.131) | 27(22.131) | 1.000 |
| hypertension, n (%) | 23(9.426) | 11(9.016) | 12(9.836) | 0.827 |
| Peripheral vascular, n (%) | 30(12.295) | 18(14.754) | 12(9.836) | 0.242 |
| Pulmonary circulation, n (%) | 15(6.148) | 9(7.377) | 6(4.918) | 0.424 |
| Valvular disease, n (%) | 9(3.689) | 5(4.098) | 4(3.279) | 0.734 |
| Cardiac arrhythmias, n (%) | 29(11.885) | 10(8.197) | 19(15.574) | 0.075 |
| BMI, body mass index | | | | |
